# Does acupuncture therapy affect peripheral inflammatory cytokines of major depressive disorder? A protocol for the systematic review and meta-analysis

**DOI:** 10.3389/fneur.2022.967965

**Published:** 2022-11-10

**Authors:** Ya-Nan Zhao, Shuai Zhang, Yu Chen, Yu Wang, Hao Chen, Yu-Ting Duan, Shao-Yuan Li, Zi-Xuan Zhang, Yi-Fei Wang, Chen Xin, Liang Li, Pei-Jing Rong

**Affiliations:** ^1^Institute of Acupuncture and Moxibustion, China Academy of Chinese Medical Sciences, Beijing, China; ^2^College of Acupuncture and Chinese Tuina, Nanjing University of Chinese Medicine, Nanjing, China; ^3^Hong Kong Chinese Medicine Clinical Study Center, School of Chinese Medicine, Hong Kong Baptist University, Kowloon Tong, Hong Kong SAR, China; ^4^Chinese EQUATOR Center, Kowloon Tong, Hong Kong SAR, China

**Keywords:** major depressive disorder, acupuncture, peripheral inflammatory cytokines, anti-inflammatory, selective serotonergic reuptake inhibitors (SSRI)

## Abstract

**Background:**

Acupuncture is widely used as adjuvant therapy for major depressive disorder (MDD). There is robust evidence that inflammation is closely associated with MDD. To date, only a few numbers of studies have investigated the potential relationship between acupuncture and the change of inflammatory biomarkers in patients with MDD. Additionally, the results are inconsistent among studies. The current study aims to provide a comprehensive, systematic review of the association between acupuncture and changes in peripheral inflammation of patients with MDD, and clarify the alterations of inflammatory cytokines before and after acupuncture treatment by meta-analysis.

**Methods and analysis:**

This study will be conducted in accordance with the Preferred Reporting Items for Systematic Reviews and Meta-Analyses guidelines. Eligible randomized controlled trials (RCTs) reporting acupuncture, with inflammatory cytokines as the outcome measured before and after intervention in patients with MDD, were searched in electronic databases, such as PubMed, Embase, Cochrane, SINOMED, Wanfang, China national knowledge infrastructure (CNKI), and Chongqing VIP (CQVIP). Primary outcomes of interest will be validated to measure the levels of inflammatory cytokines before and after acupuncture treatment in patients with MDD.

**Discussion:**

Acupuncture can drive anti-inflammatory effects, as well as symptom changes in MDD, which may represent a viable, multi-faceted treatment approach in MDD.

**Systematic review registration:**

[PROSPERO], identifier [CRD42021289207 on 04 December 2021].

## Background

Major depressive disorder (MDD) is a common mental illness, with more than 264 million people affected (1). Inflammatory cytokines are thought to contribute to the pathogenesis of MDD (2). More precisely, it is supposed that cytokines could be responsible for changes in the brain's circuits and neurotransmitter systems and consequently for behavioral changes in MDD (3). Both preclinical and clinical studies manifested that significantly higher concentrations of peripheral pro-inflammatory cytokines were found in animal depression models and patients with MDD (4). According to a recent meta-analysis (5), levels of interleukin-6 (IL-6), tumor necrosis factor-α (TNF-α), and interleukin-1 (IL-1) significantly increased in patients with MDD. Three other meta-analyses have also shown that inflammatory marker levels, such as high sensitivity C-reactive protein (hs-CRP) and IL-1, increased in patients with MDD (6–8). These findings facilitate the hypothesis of inflammation during the depression and predict that inflammation plays a role in the formation, progression, and perpetuation of MDD.

Acupuncture is one of the most popular complementary therapies. Accumulating evidence shows that acupuncture alone or combined with SSRIs/SNRIs have been more and more widely used in the field of antidepressant therapy and have embodied the double advantages of effectiveness and safety (9, 10). Acupuncture involves inserting thin and sterile needles into the skin at acupoints. Numerous meta-analyses have evaluated acupuncture-related strategies to treat MDD (11–14).

Recent studies illustrated that acupuncture may also contribute to the reduction of chronic low-grade peripheral inflammation, which is similar to antidepressant medication (15). Results from animal experiments manifested that there was a multitarget antidepressant effect of acupuncture, which may be related to inflammatory pathways (16). Mounting evidence shows that they have an anti-inflammatory effect, which is important for their therapeutic effects on anti-depression (6). Meanwhile, growing evidence supporting an inflammatory etiology of MDD has led to numerous studies which have evaluated the anti-inflammatory efficacy of acupuncture. A study reported that abdominal acupuncture stimulation amplifies an endogenous anti-inflammatory system mediated by NPYDBH-marked splenic noradrenergic neurons (17). Somatosensory autonomic reflexes allow acupuncture to modulate body physiology at distant sites (e.g., suppressing severe systemic inflammation) (18). Results from animal experiments manifested that there is a multitarget antidepressant effect of acupuncture, which may be related to inflammatory pathways (16, 19).

However, the underlying biological processes of acupuncture effects on MDD are still not fully depicted and remain inconsistent. Therefore, it is worth conducting a systematic review and meta-analysis to summarize the most updated research results for the role of acupuncture treatment in the inflammation of MDD.

## Aims and objectives

We conducted a meta-analysis using data from all relevant randomized controlled trials (RCTs) that compared the peripheral inflammatory cytokines of acupuncture in treating MDD. The purpose of this study is to provide evidence that acupuncture can be used as an adjunctive treatment to anti-depressants, which may be associated with increased anti-inflammatory effects. From the previous evidence, we hypothesized that the levels of hs-CRP, IL-1, IL-6, IL-10, and TNF-α in serum are decreased o increases in MDD individuals with acupuncture treatment by meta-analysis.

## Methods

### Search strategy

**Figure 1:**
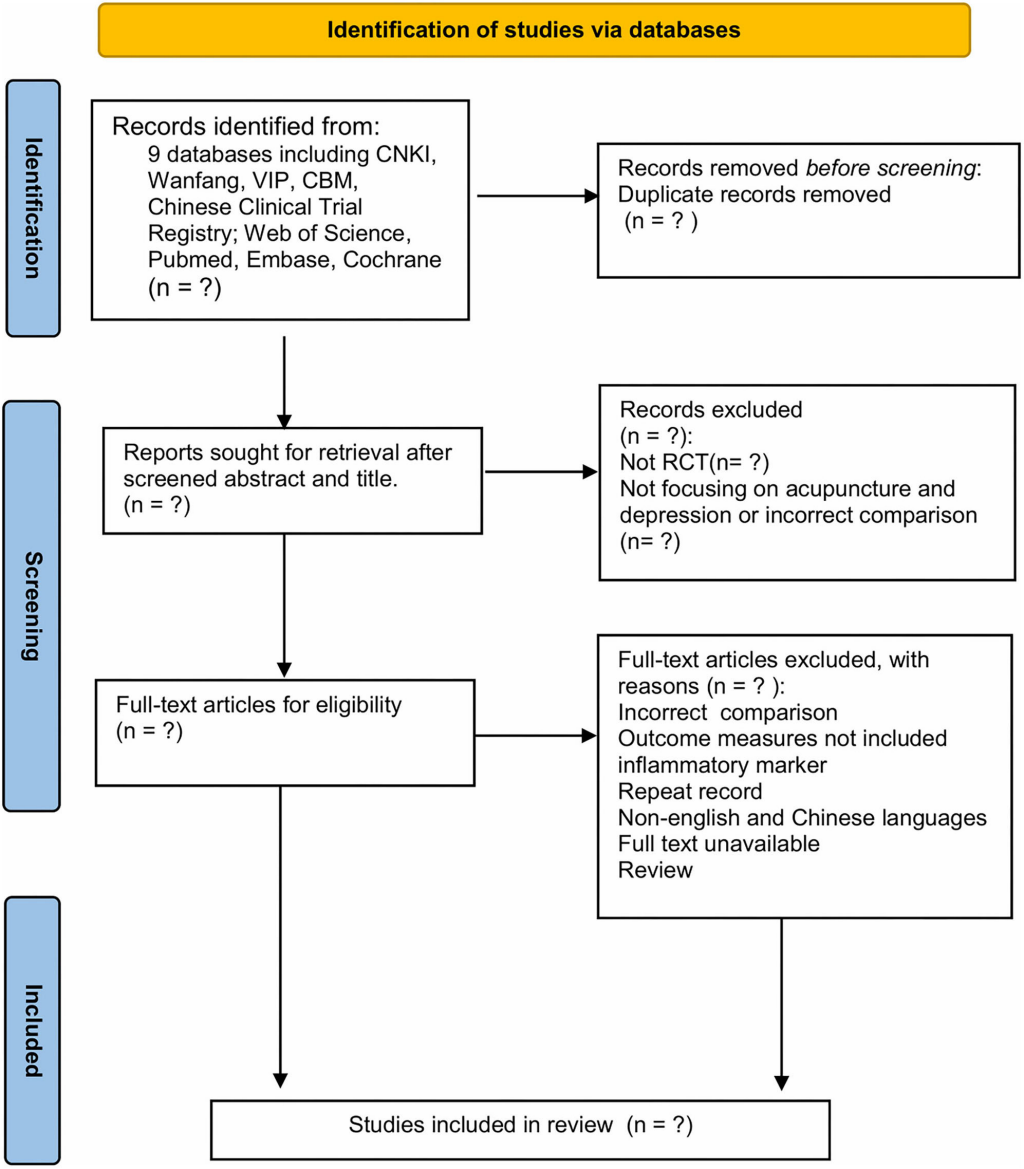
PRISMA flow diagram of the study selection process. PRISMA, Preferred Reporting Items for Systematic Reviews and Meta-Analyses.

The systematic review and meta-analysis will be conducted and reported in accordance with the Preferred Reporting Items for Systematic Reviews and Meta-Analyses (PRISMA) guidelines (20). Figure 1 summarizes the study selection as a PRISMA flowchart.

### Search methods for identification

We searched the following electronic databases: five Chinese electronic databases: SINOMED, Wanfang database, CNKI, CQVIP, and Chinese Clinical Trial Registry and four English electronic databases: Cochrane Library, Medline (*via* PubMed), Web of Science, and Embase from their inception to November 2021. The search was restricted to English- and Chinese-language studies. The search strategy combined medical subject headings (MeSH) terms with keywords. To ensure the comprehensiveness of the search, we included all clinical studies of acupuncture on MDD for screening. The search strategy of PubMed is shown in Table 1.

**Table 1:** Search strategy for PubMed.

**Search number**	**Query**
#1	“major depression” [Title/Abstract] OR “major depressive disorder” [Title/Abstract] OR “depressive symptom*” [Title/Abstract] OR “symptom, depressive” [Title/Abstract] OR “depress*” [Title/Abstract] OR “dysphor*” [Title/Abstract] OR “dysthym*” [Title/Abstract] OR “adjustment disorder*” [Title/Abstract] OR “mood disorder*” [Title/Abstract] OR “affective disorder” [Title/Abstract] OR “emotional depression*” [Title/Abstract]
#2	“Depression” [Mesh] OR “Depressive Disorder” [Mesh] OR “Depressive Disorder, Treatment-Resistant” [Mesh] OR “Depressive Disorder, Major” [Mesh]
#3	#1 OR #2
#4	“auricular acupuncture” [Title/Abstract] OR “electroacupuncture” [Title/Abstract] OR “hand acupuncture” [Title/Abstract] OR “acupuncture therapy” [Title/Abstract] OR “auriculotherapy” [Title/Abstract]
#5	“acupuncture” [Mesh]
#6	#4 OR #5
#7	#3 and #6

### Selection criteria

The studies conducting the within-group comparisons of the peripheral levels of cytokines and chemokines in patients with MDD at baseline and after acupuncture will be included in the current meta-analysis.

### Types of studies

A study with a longitudinal design including RCTs with double-blind, single-blind, or non-blind designs in English and Chinese will be included. Animal studies or studies with incomplete data will be excluded.

### Participants

Adult patients of both sexes with MDD as confirmed by the Diagnostic and Statistical Manual (DSM-III/DSM-IV/DSM-V) (21–23), the International Classification of Disease (ICD-10) (24), and the Criteria for Classification and Diagnosis of Mental Diseases (CCMD-2/CCMD-3) (24, 25) without major physical illness (diabetes, heart disease, cancer, etc.).

### Types of interventions

Interventions with acupuncture vs. sham acupuncture, acupuncture combined with SSRIs/SNRIs vs. SSRIs/SNRIs alone, acupuncture vs. SSRIs/SNRIs, and acupuncture vs. psychological intervention in an acute treatment phase (treatment duration between 4 and 12 weeks) of an MDD episode.

### Types of outcome measures

#### Primary outcomes

- Inflammatory cytokines: IL-1, IL-2, IL-4, IL-6, IL-8, IL-10, hs-CRP, TNF-α, and IFN-γ in peripheral blood points. When multiple data points were available post-intervention (pre-treatment, post-treatment, or follow-up), post-treatment data were used as the primary time point;
- Severity of MDD measured on HAMD/MADRS scale.

#### Secondary outcomes

- QoL: evaluated mainly by the Medical Outcomes Study Short Form 36;
- Safety: the adverse events measured on SERS.

### Study selection and data extraction

The outcome of this meta-analysis will be the changes in inflammatory cytokines before and after acupuncture treatment in patients with MDD, and their concentrations were measured by the standard mean differences (SMDs). EndNote V.X8.2 will be used to manage studies. First, duplicate literature will be excluded by electronic-based and manual-based steps in EndNote. Second, two reviewers will independently screen the titles and abstracts and select the studies which meet the eligibility criteria. If there are disagreements, the third reviewer will be consulted. The evaluators will read the full text of the included literature and then preliminarily extract the relevant data, mainly including the following information: first author, publication year, study design, sample size/female, mean age, gender, diagnostic criteria, acupuncture treatment (frequency, each session duration, total period, and acupoints protocol), control treatment, and inflammatory cytokines.

### Assessment of the risk of bias

According to the Cochrane Handbook for Systematic Reviews of Interventions, version 6 (26), the risk of bias 2.0 (ROB 2.0) tool will be used to mean the methodological quality and the risk of bias of the included studies. One researcher assessed the ROB of included studies by using ROB 2.0 and another researcher confirmed the judgment. If there are any differences, the third researcher will be asked to solve the problem.

### Quality assessment

The quality of evidence for main outcomes will be assessed by the Grades of Recommendation, Assessment, Development, and Evaluation (GRADE) approach. Two reviewers will do this independently through GRADEpro Guideline Development Tool. GRADE approach provides guidance for rating the quality of evidence and grading the strength of recommendations for healthcare. There are four quality levels: high, moderate, low, and very low. We also assessed the reporting quality by using the CONSORT and the STRICTA by two investigators.

### Publication bias

STATA V.14.0 will be used to evaluate publication bias. Begg's test and Egger's test will be used to assess the publication bias of the included trials and form the publication bias plot.

### Assessment of heterogeneity and sensitivity analysis

We will use the *I*^2^ statistic to assess the heterogeneity. If the *I*^2^ value is below 50%, the fixed effect model will be used. Otherwise, sensitivity analysis will be conducted to explore the main sources of heterogeneity, after which, the random effect model will be used if the *I*^2^ is still equal to or >50%. Both types of effect sizes will be presented with 95% Cis, and *p*-values < 0.05 will be regarded as statistically significant.

### Subgroup analysis

Subgroup analysis will also be conducted to explore the main sources of heterogeneity. We performed four subgroup analyses according to the frequency of treatment (once a day vs. every other day), study duration (4 vs. 8 weeks), treatment protocol of acupuncture points (semi-standardized vs. fixed-standardized), and types of antidepressant medications (SSRIs vs. SNRIs) to explore their impact on the levels of peripheral inflammatory cytokines.

### Statistical analysis

We will use the Review Manager software provided by the Cochrane Collection (RevMan5.4.1). The SMD with 95% CI will be used for continuous outcomes and not actual mean differences to measure effect sizes. While a *P*-value < 0.05 was considered statistically significant. The extent of heterogeneity will be assessed using the Chi-square test and the Higgins *I*^2^-test. A value of *P*< 0.10 or *I*^2^ > 50% indicated that the heterogeneity of effect estimates within each group of studies will be statistically significant. As the potential clinical heterogeneity among the included studies, the random effects model will be used to pool the studies. All two-tailed *P* < 0.05 will be defined as statistical significance.

### Patient and public involvement

Patients will be not involved in the development of this systematic review protocol. The data for this systematic review will be collected from previously published studies.

### Ethics and dissemination

Formal ethical approval is not required, as primary data will not be collected with the systematic review and meta-analysis. Data from previously published studies will be retrieved and analyzed. This study including protocol development will be conducted from November 2021 to October 2022. The results will be disseminated through a peer-reviewed publication and inform the most up-to-date evidence of the roles of acupuncture treatment for MDD.

## Discussion

Our current systematic review and meta-analysis will provide comprehensive evidence for the association between acupuncture and the inflammation of individuals with depression. We will use the PRISMA guidelines and checklist in the publication process. The quantitative data will be summarized and presented in tables, forest plots, and charts. The alterations of inflammatory cytokines pre- and post-acupuncture treatment in patients with MDD will be presented.

A “dose” of acupuncture is made up of multiple components. The exact components of a dose differ slightly between acupuncturists but consist of: (a) a neurophysiological dose, which includes the retention time and the treatment protocol of acupoints (fixed, semi-standardized, and individualized acupuncture) and (b) a cumulative dose, made up of the frequency (once a day, every other day, etc.) and total treatment duration (4, 6 weeks, etc.) (27–29). These multiple components not only affect the effect but also potentially affect the level of peripheral inflammatory cytokines in patients with MDD (18). Furthermore, there remain many unanswered questions concerning what might be considered optimal acupuncture.

The current study is anticipated to have some limitations. First, we might not find a sufficient number of original studies to perform the analyses. Second, the potential high heterogeneity between studies in the exposure of interest and restriction to studies in English- and Chinese-language will lead to selection bias and also decrease the reliability of our results. Third, the treatment schedule, such as the number of acupoints and frequency of treatment, will vary among studies. Fourth, large-scale studies will be required to yield stable and consistent effect sizes.

To the best of our knowledge, this will be the first meta-analysis exploring the association between acupuncture and peripheral inflammation of individuals with MDD. From our findings, we can provide the latest evidence to assist in decision-making among patients, caregivers, and clinicians in treating patients with MDD by acupuncture and as a foundation for future studies.

## Data availability statement

The original contributions presented in the study are included in the article/Supplementary material, further inquiries can be directed to the corresponding author.

## Author contributions

Y-NZ and P-JR had the idea for the article. SZ, YC, and Z-XZ performed the literature search. Y-NZ and SZ drafted the manuscript. P-JR and YW critically revised the work. Y-TD, HC, and S-YL helped conceptualize the study. CX and LL embellished the language of the manuscript. Y-FW provided valuable advice on writing the manuscript. All authors read and approved the final manuscript.

## Funding

This study was supported by the National Key R&D Program of China (No. 2018YFC1705800), the Acupuncture and Chronobiology Key Laboratory of Sichuan Province (No. 2021004), and the Fundamental Research Funds for the Central public welfare research institutes (ZZ15-YQ-048).

## Conflict of interest

The authors declare that the research was conducted in the absence of any commercial or financial relationships that could be construed as a potential conflict of interest.

## Publisher's note

All claims expressed in this article are solely those of the authors and do not necessarily represent those of their affiliated organizations, or those of the publisher, the editors and the reviewers. Any product that may be evaluated in this article, or claim that may be made by its manufacturer, is not guaranteed or endorsed by the publisher.
