# Supplementary material for: Does acupuncture therapy affect peripheral inflammatory cytokines of major depressive disorder? A protocol for the systematic review and meta-analysis
Source: Front Neurol. 2022 Nov 10;13:967965. doi: 10.3389/fneur.2022.967965 (PMC9685430; doi:10.3389/fneur.2022.967965)
Supplement: Supplementary file 1 [file Data_Sheet_1.docx]

**Supplementary File 1. Search strategy for database.**

**Database Search strategy**

**PubMed Website: https://www.ncbi.nlm.nih gov/pubmed**

(((((((((acupuncture[MeSH Terms]) OR (auricular acupuncture[Title/Abstract])) OR (electroacupuncture[Title/Abstract])) OR (hand acupuncture[Title/Abstract])) OR OR (acupuncture therapy[Title/Abstract])) OR (auriculotherapy[Title/Abstract])) AND (((((((((depression[MeSH Terms]) OR (Depressive Symptom[Title/Abstract])) OR (depressive disease[Title/Abstract])) OR (depressive episode[Title/Abstract])) OR (parental depression[Title/Abstract])) OR (melancholia[Title/Abstract])) OR (mourning syndrome[Title/Abstract])) OR (affective disorder[Title/Abstract])) OR (treatment resistant depression[Title/Abstract])

**Web of Science Website:https://www.webofscience.com**

#1 TS=(depressive disorder OR Depressive Disorders OR Disorder, Depressive OR Disorders, Depressive OR Neurosis, Depressive OR Depressive Neuroses OR Depressive Neurosis OR Neuroses, Depressive OR Depression, Endogenous OR Depressions, Endogenous OR Endogenous Depression OR Endogenous Depressions OR Depressive Syndrome OR Depressive Syndromes OR Syndrome, Depressive OR Syndromes, Depressive OR Depression, Neurotic OR Depressions, Neurotic OR Neurotic Depression OR Neurotic Depressions OR Melancholia OR Melancholias OR Unipolar Depression OR Depression, Unipolar OR Depressions, Unipolar OR Unipolar Depressions )

#2 TS=(scalp acupuncture OR scalp needle OR scalp electroacupuncture OR eye acupuncture OR eye needle OR abdominal acupuncture OR abdominal needle OR ear acupuncture OR ear needle OR auricular acupuncture OR auricular needle)

#3 TS=(Randomized Controlled Trial OR controlled clinical trial OR randomized OR random OR controlled trial OR randomly)

#4 #1 AND #2 AND #3

**Embase Website:** [**https://www.embase.com/**](https://www.embase.com/)

#1'acupuncture'/exp OR 'auricular acupuncture':ti,ab,kw OR electroacupuncture:ti,ab,kw OR 'hand acupuncture':ti,ab,kw OR 'acupuncture therapy':ti,ab,kw OR auriculotherapy:ti,ab,kw

#2 'depression'/exp OR 'depressive disease':ti,ab,kw OR 'depressive episode':ti,ab,kw OR depression:ti,ab,kw OR 'depressive symptom':ti,ab,kw OR 'mental depression':ti,ab,kw OR 'parental depression':ti,ab,kw OR melancholia:ti,ab,kw OR 'mourning syndrome':ti,ab,kw OR 'affective disorder':ti,ab,kw OR 'treatment resistant depression':ti,ab,kw

#1 AND #2

**Cochrane (CENTRAL) Website:https://www.cochranelibrary.com/**

#1 MeSh descriptor: [acupuncture] explode all trees

#2 acupuncture:ti,ab,kw OR electroacupuncture:ti,ab,kw OR hand acupuncture:ti,ab,kw OR auricular needle:ti,ab,kw OR auriculotherapy:ti,ab,kw

#3 MeSh descriptor: [depression] explode all trees

#4 “depression”:ti,ab,kw OR “depressive disorder”:ti,ab,kw

#5 #1 OR #2

#6 #3 OR #4

#7 #5 AND #6

**CNKI Website: http:/www.cnki.net**

(SU=('针刺'+'电针'+'体针'+'耳针'+'头针'+'毫针'+'针刺治疗'+'针灸疗法') OR TI=('针刺'+'电针'+'体针'+'耳针'+'头针'+'毫针'+'针刺治疗'+'针灸疗法') OR KY=('针刺'+'电针'+'体针'+'耳针'+'头针'+'毫针'+'针刺治疗'+'针灸疗法') OR AB=('针刺'+'电针'+'体针'+'耳针'+'头针'+'毫针'+'针刺治疗'+'针灸疗法')) AND (SU=('抑郁症'+'抑郁'+'郁症'+'郁病'+'忧郁症') OR TI=('抑郁症'+'抑郁'+'郁症'+'郁病'+'忧郁症') OR KY=('抑郁症'+'抑郁'+'郁症'+'郁病'+'忧郁症') OR AB=('抑郁症'+'抑郁'+'郁症'+'郁病'+'忧郁症'))

**Wanfang Website: http://www.wanfangdata.com.cn/index.html**

((主题:”针刺” or 题名或关键词:”针刺”or 摘要:”针刺” or 主题:"电针" or 题名或关键词:"电针" or 摘要:"电针" or 主题:"耳针" or 题名或关键词:"耳针"or 摘要:"耳针" or 主题:"耳廓针刺" or 题名或关键词:"耳廓针刺" or 摘要:"耳廓针刺" or 主题:"头针" or 题名或关键词:"头针" or 摘要:"头针" or 主题:"手针" or 题名或关键词:"手针" or 摘要:"手针" or 主题:"腹针" or 题名或关键词:"腹针"or 摘要:"腹针" or 主题:"毫针" or 题名或关键词:"毫针" or 摘要:"毫针") and (主题:"抑郁症" or 题名或关键词:"抑郁症"or 摘要:"抑郁症" or 主题:"抑郁" or 题名或关键词:"抑郁"or 摘要:"抑郁" or 主题:"郁症" or 题名或关键词:"郁症"or 摘要:"郁症" or 主题:"郁病" or 题名或关键词:"郁病"or 摘要:"郁病" or 主题:"忧郁症" or 题名或关键词:"忧郁症"or 摘要:"忧郁症"))

**VIP Website: http:/lib.eqvip.com**

(M=针刺 OR R=针刺 OR M=电针 OR R=电针 OR M=耳针 OR R=耳针 OR M=耳廓针刺 OR R=耳廓针刺 OR M=头针 OR R=头针 OR M=手针 OR R=手针 OR M=腹针 OR R=腹针 OR M=毫针 OR R=毫针) AND (M=抑郁症 OR R=抑郁症 OR M=抑郁 OR R=抑郁 OR M=郁症 OR R=郁症 OR M=郁病 OR R=郁病 OR M=忧郁症 OR R=忧郁症)

**CBM Website:** [**http://www.sinomed.ac.cn/**](http://www.sinomed.ac.cn/)

(("抑郁症"[常用字段:智能] OR "抑郁"[常用字段:智能] OR "郁症"[常用字段:智能] OR "郁病"[常用字段:智能] OR"忧郁症"[常用字段:智能]) AND (("针灸"[标题] OR ("针刺"[标题] OR "电针"[标题] OR "体针"[标题] OR "耳针"[标题] OR "耳针刺"[标题] OR "耳廓针刺"[标题] OR "耳廓针刺术"[标题]) OR "毫针"[标题] OR ("针刺治疗"[标题] OR "针刺疗法"[标题] OR "针灸疗法"[标题]) OR ("针灸"[摘要] OR "针刺"[摘要] OR "药物针刺"[摘要] OR "电针"[摘要] OR "体针 "[摘要] OR "耳针"[摘要] OR "耳针刺"[摘要] OR "耳廓针刺"[摘要] OR "耳廓针刺术"[摘要])OR ("毫针"[摘要]))

**Chinese Clinical Trial Registry Website:** [**http://www.chictr.org.cn/searchproj.aspx**](http://www.chictr.org.cn/searchproj.aspx)

Registered subject：抑郁症、郁症、郁病、忧郁症；针灸、电针、针刺、头针、耳针
